# Target Antigens in Western and Line Immunoblots for Supporting the Diagnosis of Lyme Disease. Comment on Porwancher et al. Immunoblot Criteria for Diagnosis of Lyme Disease: A Comparison of CDC Criteria to Alternative Interpretive Approaches. *Pathogens* 2023, *12*, 1282

**DOI:** 10.3390/pathogens13050352

**Published:** 2024-04-25

**Authors:** Jyotsna S. Shah, Ranjan Ramasamy

**Affiliations:** IGeneX, 556 Gibraltar Drive, Milpitas, CA 95035, USA

An article was recently published in *Pathogens* on using different target antigens from *Borrelia* species that cause Lyme disease for detecting serum antibodies to support a clinical diagnosis of Lyme disease (LD) [1]. This article tries to show that the antigen criteria first recommended in 1994 by the US Centers for Disease Control and Prevention (CDC) for the second-tier Western blot (WB) in standard-two tier tests (STTTs) [2], and approved by the US Food and Drug Administration (FDA), were superior to the modified WB antigen criteria used at IGeneX [1]. The establishment of the CDC antigen reactivity criteria for the second-tier WBs of STTTs in 1994 significantly advanced LD serodiagnosis in the US. We consider it important for progressing the underlying science to list some fundamental deficiencies in the *Pathogens* article cited herein as [1].

1. In the legend of Table 1, [1] states that IGeneX uses WBs as a single-tier diagnostic approach for all stages of LD, basing this on two IGeneX publications from 2010 and 2014 [3,4]. IGeneX provided first-tier enzyme immunoassays (EIAs) and fluorescent antibody tests as well as second-tier WB or immunoblot (IB) tests, as requested by physicians. Article [1] did not make it clear that WBs and IBs are always read by both in-house and CDC criteria for antigen positivity at IGeneX and both results are reported to the physicians requesting tests [1]. IGeneX reports EIA and IB results separately, stating in its report form “For diagnostic purposes, immunoblot test results should be used in conjunction with clinical symptoms and other evidence available to the diagnosing physician”.

2. The 2010 and 2014 IGeneX publications [3,4] reported important findings pertaining to the use of different types of WBs in LD diagnosis and not the use of WBs as single-tier alternative tests to STTTs as incorrectly assumed in [1]. Important findings reported by IGeneX in the two publications [3,4] were as follows: (i) the use of a mixed-cell lysate from two *Borrelia burgdorferi* sensu stricto strains, B31 and 297, improved WB sensitivity compared to lysate from a single strain; and (ii) the need to verify that the recognition of a WB band of approximate molecular mass of 31 kDa was due to OspA by a separate test with recombinant OspA as a target antigen. Excluding the recognition of non-OspA antigens of similar molecular mass to OspA in this manner improved the specificity of the IGeneX criteria to >97%, which was comparable with that obtained using the CDC criteria (which do not use OspA) on the same samples [4]. IGeneX also clearly states that a positive antibody reaction with OspA is not to be considered in persons vaccinated with OspA. Article [1] does not highlight any of this.

3. The IGeneX pilot study of 2018 [5] primarily reported the advantages of using IBs with purified *B. burgdorferi* antigens over WBs with whole-cell lysates in STTTs. This pilot study suggested that it may eventually become possible to develop single-tier IB tests for LD [5]. Article [1], however, incorrectly implies that the pilot study [5] claimed to establish a single-tier IB test for LD.

4. In its legend to Table 1, article [1] states that “recombinant antigens from 4 European and 4 North American strains or genospecies of *B. burgdorferi sensu lato*” are used in IGeneX line IBs, citing an IGeneX publication [5] which did not mention such numbers of *Borrelia* species. The corresponding statement in [1] is therefore incorrect.

5. Purified antigens from multiple species of *B. burgdorferi sensu lato* have been used in a line IB format for LD diagnosis in Europe for decades [6,7,8]. Widely used European line IBs rely on fewer antigens and antigens from different *B. burgdorferi sensu lato* species, and the antigen criteria for positivity are therefore markedly different from the CDC-recommended criteria in the US [6,7,8]. There is suggestive serological evidence for *Borrelia garinii* and *Borrelia afzellii* infections in Mexico [9]. *Borrelia garinii* has been identified by DNA sequencing in mice from LD-endemic areas of the US [10]. Importantly, US residents can acquire *B. burgdorferi sensu lato* infections when they travel abroad. IGeneX IBs therefore use relevant target antigens from different *B. burgdorferi sensu lato* species but not in the incorrect format reported in [1]. The negative assessment of IGeneX criteria in [1] ignores these considerations.

6. The use of WBs, as exhaustively analyzed and interpreted in [1], is mostly irrelevant in today’s context in Europe and the US because WBs have been largely replaced with IBs. The IGeneX criteria for line IB positivity and target antigen use in line IBs are continuously refined and have now been standardized with a much larger number of serum samples from the CDC Lyme Serum Repository (LSR) and other serum collections. However, the 2018 IGeneX pilot study [5] is helpful in that it is the only publication to this day to demonstrate, by direct parallel experimental testing, that the sensitivities and specificities when applying the CDC and IGeneX antigen criteria for IBs were similar, and also comparable to those obtained with STTTs utilizing EIAs as first-tier tests and WBs as second-tier tests on the same set of samples. This seminal experimental finding is ignored in the theoretical meta-analyses presented in [1].

7. An important point not made clear in [1] is that when antigens from different *B. burdorferi sensu lato* species are used, for example, with Osp C, a reaction with any one of the homologous antigens is considered positive for that antigen when applying the CDC and IGeneX criteria to IBs analyzed in [5]. Furthermore, line IB tests at IGeneX always include a calibration control that determines the threshold for reporting antigen reactivity as positive. Although this is clearly stated in [5], it was wrongly reported to be otherwise in [1], on page 5, paragraph 1.

8. The article [1] negatively assesses IGeneX criteria utilizing eight datasets listed in Table 3. Of these, dataset no. 8 was obtained through a private material transfer agreement between one of the authors and the CDC with the original details of antigen reactivity not available to readers. Furthermore, antibody reactivity with OspA and OspB was not available in dataset no. 8 and therefore did not include data for all interpretative criteria. Six of the eight datasets only had summary data accessible through the FDA link provided in [1]. For one of these (Immunetics IgG WBs, dataset no.1), individual data on reactivity with OspA and OspB are shown in the article [1] in Supplementary File S3, but the original data are inaccessible to readers. Dataset 4 is derived from a published article that had information on OspA and OspB reactivity, but the data used in [1] are stated to be a selection of the published data. There is theoretical evidence in [1] to suggest that the specificities of the CDC and IGeneX antigen criteria in WBs and IBs may be comparable on MarDx Marblot WBs (Tables 5,6), as also experimentally shown in [5], but article [1] employs uncommonly complex statistical analyses to try and suggest otherwise. We cite one example from its Supplementary file S2, page 4, para 2, that like many others in [1] is not readily comprehensible to readers—“Although results for alternative Criteria A (the IGeneX criteria) and B were not available for this validation subset, results for alternative criteria are reported using the entire LSR; it is assumed that this validation subset constitutes a representative sample of the overall LSR dataset”.

9. In view of the above, it is obviously disconcerting that the declarations in [1] reveal the conflicting commercial interests of its authors.

10. Parallel experimental testing of sera in IBs by the in-house IGeneX and CDC criteria will resolve contentious issues raised in [1] and advance the science behind LD serodiagnosis for the public good. We will gladly facilitate this.
